# Value-Based Healthcare as a Competitive Strategy—A Multi-Stakeholder Perception Analysis in Portuguese Healthcare

**DOI:** 10.3390/jmahp13030044

**Published:** 2025-09-02

**Authors:** Filipe Santiago, Filipe Costa, Eduardo Redondo, Cristiano Matos

**Affiliations:** 1 Medtronic, 1600-209 Lisbon, Portugal; 2 Nova School of Business and Economics, Nova SBE Carcavelos Campus, 2775-405 Carcavelos, Portugal; filipe.costa@novasbe.pt (F.C.); eduardoredondo@hotmail.com (E.R.); 3 Quinta do Bom Nome Campus, European University, 1500-210 Lisbon, Portugal; 4 Polytechnic Institute of Coimbra, Coimbra Health School, Farmácia, 3046-854 Coimbra, Portugal; cristiano.matos@estesc.ipc.pt

**Keywords:** value, healthcare, financing, value-based healthcare, competitive strategy, stakeholders, perception, qualitative study

## Abstract

Designing an accessible, financially viable healthcare system is a key challenge for society. The value-based healthcare (VBHC) strategic model aims to simultaneously improve the quality of healthcare and the efficiency of health systems. The aim of this research was to describe the perceptions of different stakeholders in the Portuguese health industry about the creation of value and the understanding of VBHC as a competitive advantage. A qualitative study was conducted using the inductive method of Braun and Clarke, designed according to the COREQ criteria. Based on the results of the literature review, a semi-structured script for an interview was created, consisting of eight questions. The initial interview script was based on a thorough narrative literature review and tested with two professionals with practical experience in VBHC. The final version of the semi-structured interview guide consisted of eight open-ended questions. The questions were designed to elicit in-depth, reflective responses, and their neutrality was reviewed to avoid leading language that might introduce bias. As the interviews progressed, minor iterative changes were made to include participant-suggested additions, always maintaining alignment with the research objectives. This iterative process was essential to capture the nuanced perspectives of stakeholders and conformed to COREQ standards for qualitative research. A total of 15 stakeholders in VBHC were interviewed. The interviews were transcribed and coded, and 605 codes were created, divided into subthemes and themes. VBHC implementation faces several challenges, requiring a collaborative effort by the stakeholders involved, to achieve a comprehensive vision of value and appropriate multi-stakeholder alignment. The implementation of VBHC can confer a sustainable competitive advantage, and its adoption as a strategic model will be inevitable in the future.

## 1. Introduction

Worldwide, different health systems are experimenting with and testing value-based payment models, replacing or changing traditional fee-for-service models that tie reimbursement to quality and cost [1]. Value-Based Healthcare (VBHC) is considered one solution to the financial pressures that healthcare systems experience [2], focusing on patient outcomes while considering the costs of achieving those outcomes [3]. It is based on the principles of maximizing value for the patient by delivering healthcare that is organized around the patient’s medical conditions and complete care cycles, while also assessing both health outcomes and the costs associated with achieving those results [4]. Different authors emphasize the need for a shift in healthcare systems to improve efficiency and patient outcomes. Porter et al. highlighted that health systems around the world are facing rising costs and inconsistent quality, advocating for a move away from a traditional, procedure-driven approach to a patient-centered system organized around patients’ needs [5]. Costa et al. supported this perspective, arguing that VBHC systems can maintain a dynamic balance among various forces and deliver high-quality care, leading to significant improvements in quality of life at sustainable costs [6]. Such a system is advocated as being sustainable in the long term, since it generates resource savings and learning opportunities that are systematically reinvested in new care cycles, contributing to the sustained creation of value and the economic viability of this system [6].

Despite the recognized importance of value in healthcare, it remains a concept that is often undermeasured and not fully understood [7]. Value in healthcare goes beyond simple financial metrics; it includes improved health outcomes [8], efficient use of resources [9], personalized care, and respect for patient preferences [10]. A comprehensive understanding of value must include an integrated perspective that takes into account the benefits for all stakeholders involved in the healthcare process [5].

Strengthening the healthcare value chain requires collaboration among diverse stakeholders, including government entities, third-party payers, patient communities, healthcare providers, suppliers, and society at large. To effectively co-create VBHC solutions, stakeholders need to approach these partnerships as investments, developing holistic, integrated solutions so they can better manage the value chain, which contributes to societal sustainability, improves patient outcomes, and generates tangible benefits for all involved, aligning with their business models [3]. This collaborative framework is underpinned by stakeholder theory, which highlights the need to engage and align the interests of all parties to ensure the sustainable co-creation of value within the healthcare ecosystem. Stakeholder theory has undergone significant development since its introduction in the 1960s, evolving as a response to the shareholder primacy model, which focused solely on maximizing shareholder value [11,12]. Pioneers like Freeman (1984) expanded this perspective, arguing that a firm’s responsibilities encompass not just shareholders, but also a wider array of stakeholders, including customers, employees, suppliers, communities, and even future generations [13,14]. Mitchell et al.’s (1997) salience theory further aids firms in prioritizing stakeholder claims by assessing power, legitimacy, and urgency [14].

Building on this idea, Preble (2005) emphasized that organizations must recognize their mutual dependencies to ensure long-term success [15]. The author outlined a six-step model of stakeholder management, as follows: stakeholder identification, understanding stakeholder claims, determining performance gaps, prioritizing demands based on salience, developing organizational responses, and ongoing monitoring and control [15], which reflects how stakeholder theory can be operationalized within organizations [15].

The practical application of stakeholder theory is highlighted in the stakeholder management cycle framework [14]. The first step, stakeholder identification, is critical for recognizing which groups affect or are affected by the organization’s actions, which aligns with Preble’s model, where identifying relevant stakeholders is foundational to any stakeholder management process. Following this, the general nature of stakeholders’ claims must be understood, which includes evaluating the different types of power they hold—whether political, economic, legal, or informational.

Stakeholder theory emphasizes the importance of recognizing performance gaps, which highlight the discrepancy between what stakeholders expect and what the organization is delivering. This gap analysis is crucial for firms to address stakeholder demands effectively. Furthermore, stakeholder salience is explored in depth, helping firms prioritize demands based on their power, urgency, and legitimacy. Mitchell et al.’s framework supports this step, aiding organizations in deciding which stakeholders to address immediately and which to manage over time [14].

Once stakeholders are prioritized, firms must develop organizational responses, which can range from broad strategies like mission alignment to more direct tactics like negotiation or partnership. For example, companies can integrate stakeholder concerns into their mission statements or engage in direct communication to resolve conflicts [15].

Finally, monitoring and control ensure that the organization’s approach remains aligned with stakeholder needs over time. A stakeholder map featured in presentations is a practical tool that helps visualize these relationships, showing connections, potential blockages, and opportunities for influence. This map helps firms maintain an adaptive and responsive approach to their stakeholder environment [15].

The gradual change in the conceptual paradigm of health systems and the necessary strategic repositioning of stakeholders in the health industry in Portugal have led to the need to understand whether stakeholders consider that they will be able to obtain a sustainable competitive advantage over their competition with the implementation of VBHC.

Considering the complexity of multiple stakeholders, each with their own and often conflicting objectives, and the conceptual ambiguity surrounding the VBHC model, it is crucial to understand each stakeholder’s perspective on what they deem essential for effective alignment in the co-creation of value. The aim of this research was to understand the perceptions of different stakeholders in the Portuguese health industry about the creation of value and the understanding of VBHC as a competitive advantage.

## 2. Materials and Methods

Qualitative research was carried out using an inductive method, with the aim of deepening the concepts, perceptions, and experiences of the different stakeholders under analysis [16,17]. The research was designed in accordance with the consolidated criteria for reporting qualitative research, COREQ [18].

A semi-structured guide was prepared for interviews. The script initially contained 8 open-ended questions to allow the interviewee to provide in-depth answers related to VBHC—Supplementary Table S1. Interviews were performed from April 2023. Through suggestions proposed in first interviews, some questions were changed, simplifying the language and improving the order of the questions, so that the interview became more fluid in its application. The script was updated throughout the interviews, including adding questions that the interviewees considered relevant. Data collection took place through individual remote interviews, lasting approximately 30–40 min each, allowing the researcher to delve deeper into the participants’ perceptions and experiences.

### 2.1. Selection of Participants

Participants were selected through a combination of convenience and snowball sampling, with the goal of including a heterogeneous mix of stakeholders with direct experience or leadership roles in VBHC initiatives in Portugal. Initially, stakeholders were selected through direct contact with individuals known to the researcher and recognized for their experience and relevance to the research topic (convenience sampling). Subsequently, other participants were recruited through snowball sampling [19] to ensure the selection of participants who were particularly knowledgeable, directly involved, or interested in VBHC, also ensuring the inclusion of individuals with a high level of knowledge and expertise, providing a valuable perspective on the involvement of different stakeholders in VBHC in Portugal.

A total of 18 stakeholders were approached, of whom 15 agreed to participate, resulting in an 83% response rate. The final sample included representatives from patient associations, public and private healthcare providers, pharmaceutical and medical device suppliers, public and private payers, academic researchers, members of healthcare consortia, and consultants. Their organizational types, nationalities, roles, and sex (where disclosed) are detailed in Supplementary Table S2.

### 2.2. Recruitment Process

An email invitation was sent to potential interviewees, which included a formal invitation letter together with an informed consent form in accordance with EU general data protection regulations [20]. If the first invitation e-mail was unanswered, two reminder e-mails were sent at 2-week intervals. Participants who agreed to participate scheduled an interview with the main researcher (F.S.), which was carried out through the platform Microsoft Teams^®^. After participant consent was obtained, the interviews were recorded and transcribed verbatim with Cockatoo ^®^ software (v1, Cockatoo Inc., Tempe, AZ, USA) and curation by the main researcher (F.S.). Interviewees’ confidentiality was ensured by anonymizing the data, assigning each participant a numerical code used to report the results. The number of interviewees was determined based on data saturation, i.e., the point at which no new codes emerged from the data after a new interview [21,22]. At the point of data saturation, the snowball process was terminated and only interviews already scheduled were completed.

### 2.3. Interview Processing and Coding

All interviews were transcribed and analyzed using Atlas.ti^®^ software (Web 5.21 version, Berlin, Germany). In vivo coding was used to retain participant language and reduce interpretation bias. Following Braun and Clarke’s (2006) thematic analysis framework, codes were grouped into subthemes and broader themes based on their relationships [17]. The first interview was independently coded by the main researcher and a VBHC expert, and differences were reconciled through discussion to enhance coding reliability. Data saturation was reached when additional interviews produced no new codes or themes; this point was actively monitored, and only interviews already scheduled were conducted thereafter [23]. Inductive analysis was used, guided by the data, without trying to fit into a pre-existing coding model or the researcher’s analytical prejudice. The codes generated by the initial analysis were then grouped based on the relationship or connection between codes, which generated high-level concepts, the subthemes. Subtheme groups were then related to each other in larger groups to create themes.

The coding process involved in vivo coding to minimize researcher interpretation bias. An independent expert in VBHC co-coded the initial transcript to ensure reliability, and discrepancies were discussed until consensus was reached [24]. The coding process remained active throughout the analysis, meaning that active modification of codes, subthemes, and themes continued until a final set of connections was clearly established.

## 3. Results

The results section provides a detailed thematic analysis of stakeholder perspectives, with quotes illustrating their views on value co-creation and competitive advantage through VBHC. The key themes explored include the challenges of multi-stakeholder alignment, the role of trust and negotiation, the importance of communication, and the need for effective evaluation systems. Additionally, stakeholders highlighted VBHC as a source of competitive advantage through differentiation, evidence-based results, continuous improvement, transparency, and better access to resources and financing.

### 3.1. Multi-Stakeholder Alignment in Value Co-Creation

The implementation of VBHC with the appropriate co-creation of value requires strong alignment between all stakeholders in the health system [25]. Given the evident heterogeneity of the different stakeholders, alignment to create value poses several challenges, requiring everyone to be committed to the same objectives, willing to negotiate and make compromises, communicate and collaborate, and also use effective metrics, monitoring, and evaluation systems—Table 1.

#### 3.1.1. Establishing Common Purpose and Trust

The successful implementation of VBHC from a perfect alignment between stakeholders to achieve value-driven results is considered a basic assumption. However, it is necessary to establish a solid foundation of trust to overcome challenges in measuring value and interpreting results. The success of VBHC and value co-creation will depend on effective collaboration and mutual trust between stakeholders in this value chain. Porter et al. stated that improving performance and accountability depends on having a shared objective that unites the interests and activities of all interested parties [5]. It is defined that the establishment of a shared purpose and trust, the alignment of interests, collective awareness, and a vision of sustainability in the health ecosystem can involve all stakeholders in a meaningful way.

#### 3.1.2. Availability of Negotiation and Risk Sharing

Implementing VBHC and value co-creation requires that all stakeholders are willing to negotiate and make compromises. In this sense, all necessary data must be available and effective intermediation must be ensured, as these are elements that facilitate the negotiation process. The focus should be on agreeing on the end goal and documenting the value delivered. Finally, risk-sharing strategies require common objectives and joint work between various stakeholders, thus creating greater alignment and a greater capacity to affect positive results.

#### 3.1.3. Effective Communication and Collaborative Momentum

In pursuing the appropriate implementation of VBHC, it is essential to have clear and effective communication between all stakeholders involved in all stages of the project. Another critical aspect mentioned refers to the collaborative impetus to learn from what others do well, demonstrating a greater sensitivity to replicating successful practices.

#### 3.1.4. Measuring and Evaluating Value Across the Entire Value Chain

Aligning the implementation of VBHC between stakeholders is considered essential to measure and evaluate the value generated by each stakeholder, identifying the most efficient level for producing this value. Stakeholders argue that the financing model must follow this value allocation.

### 3.2. VBHC as a Competitive Advantage

VBHC aims to create value through the healthcare industry value chain, taking into account patient satisfaction and good healthcare outcomes at the best cost. From the perspective of the stakeholders involved, adopting VBHC approaches allows organizations to stand out and offer significant value to patients, while also improving value for other stakeholders involved in the care process—Table 2.

#### 3.2.1. Competitive Advantage Through Differentiation

In the healthcare industry in Portugal, there is intense competition for the implementation of differentiation strategies to achieve a competitive advantage. The stakeholders involved believe that the VBHC approach can guarantee a competitive advantage through differentiation. This competitive advantage can be achieved through patient centricity and the generation of value that the model itself presupposes. A competitive advantage can also be achieved by differentiating an organization from its average or typical competitor through the ability to measure, monitor, evaluate, and compare data on health outcomes, providing greater efficiency and quality of processes. Stakeholders also understand that this generation of valuable and proven information on health outcomes is highly differentiating compared to less effective competitors without clear evidence of efficiency.

#### 3.2.2. Competitive Advantage Through Evidence of Results

From the stakeholder perspective, companies that adopt VBHC will be able to solidify their positioning, guaranteeing a competitive advantage based on evidence of the results achieved. By adopting this VBHC approach, stakeholders obtain a real, concrete, and absolute vision of the results achieved for the patient, which allows them to prove therapeutic effectiveness, ensure resource efficiency, and stand out from competition that assumes empirical strategic models, more perceived than factual.

#### 3.2.3. Competitive Advantage by Pursuing Continuous Improvement

Stakeholders emphasize that VBHC promotes potential competitive advantages by promoting opportunities for improvement in areas that are not adequately developed. They consider that through this positive progression, it will be easier to achieve significant improvements in terms of efficiency, quality, and clinical results. They state that VBHC sets up a culture of sharing and collaboration between stakeholders in the pursuit of continuous improvement, which can guarantee a competitive advantage resulting from the possibility of continuous learning. They also argue that through the ability to evaluate the impact of their decisions, they will be able to understand their effectiveness, thus allowing them to stand out from the competition by promoting quality and innovation.

#### 3.2.4. Competitive Advantage Through Transparency

For stakeholders, the comparability of success rates will become a differentiating factor for patients to choose the best option, in which transparency will enable the introduction of incentives for continuous improvements and quality services to overcome resistance to competition in the healthcare industry. This transparency could also promote solid and sustainable partnerships, which in itself represents an important sustainable competitive advantage. These highlighted aspects, when combined, could allow leading stakeholders to deliver quality services and effective results, thus providing them with a competitive advantage in the market.

#### 3.2.5. Competitive Advantage Through Easier Access to Resources and Financing

According to the responses received, implementing VBHC projects can promote distinction. The distribution of financing based on value metrics clarifies the scope of the intended results, making the allocation of resources fairer and more efficient. The adoption of VBHC also constitutes a competitive advantage by increasing the possibility of obtaining financing and ensuring a more strategic and results-oriented allocation.

#### 3.2.6. Competitive Advantage Through Competitiveness and Reputation

Despite the current limitations in comparability, the competitiveness driven by VBHC must be based on the effectiveness and efficiency of the services provided by stakeholders. These are key factors in differentiating and attracting more customers. Furthermore, involvement in VBHC projects will generate a competitive advantage in itself, as credibility and reputation play a significant role in the decisions made by patients and other stakeholders.

## 4. Discussion

VBHC is a healthcare delivery model that has the potential to improve outcomes for patients while simultaneously adjusting their healthcare costs [3]. However, the model requires strong alignment among different healthcare system stakeholders. In order to achieve this, all stakeholders must be committed to the same objectives, willing to negotiate and make compromises, communicate and collaborate effectively, and utilize robust metrics, monitoring, and evaluation systems.

This study explored how the principles of VBHC, as globally advocated, are understood and evaluated by stakeholders within the Portuguese healthcare system. While the theoretical benefits of VBHC are broadly recognized across countries—such as improving patient outcomes, promoting efficiency, and enhancing transparency—our findings indicate that the Portuguese healthcare environment presents unique contextual challenges and opportunities that shape its implementation trajectory. Although the foundational principles of VBHC have universal relevance, their practical application must be tailored to regional realities; in Portugal, the uneven distribution of healthcare resources underscores the importance of adapting implementation strategies to ensure both efficiency and equity. The Portuguese healthcare system is structured around the Serviço Nacional de Saúde (SNS), a publicly funded, universal-access model managed centrally by the Ministry of Health. Healthcare financing is primarily sourced from general taxation, with additional contributions from user fees and voluntary health insurance. Service delivery is organized through regional health administrations, and while the public sector dominates, there is an increasing presence of private providers [26,27,28] To clarify the specific challenges in applying VBHC in Portugal, Table 3 presents the key barriers identified during the study and why they are particularly relevant within the national healthcare context.

The theme of multi-stakeholder alignment emerged as particularly complex in Portugal due to entrenched institutional silos, varying definitions of value, and limited trust between healthcare actors. Stakeholders consistently emphasized that the success of VBHC depends on establishing a shared purpose, grounded in trust and collective vision. A hospital provider remarked, “*A common purpose, alignment of trust, shared awareness, and a joint sustainability vision in the health ecosystem could increase involvement from all stakeholders*” (Provider 2.3.62). This view was echoed by a consortium member, who described “*perfect alignment between stakeholders*” as a basic prerequisite for any VBHC strategy (Consortium 2.12.1). However, stakeholders also highlighted the challenges in aligning objectives and expectations, particularly in the context of negotiation and risk sharing. As one provider noted, “*There must be negotiation and compromise*” (Provider 2.2.51), while a supplier emphasized that effective data sharing is crucial to enable these processes, “*As long as we agree on the final end and the forms of data collection that allow me to document value, I can’t question that*” (Supplier 2.9.42). These insights reveal the tension between theoretical alignment and practical execution, particularly when risk-sharing mechanisms require a foundation of reliable data and mutual trust. Communication and collaborative momentum were also viewed as essential enablers. A provider stressed the importance of involving all actors from the outset and ensuring “*clear and effective communication at all phases of the project*” (Provider 2.2.52). Additionally, stakeholders called for greater sensitivity to replicating successful practices, as expressed by a patient representative, “*We should be more sensitive to replicating what others do well*” (Consumer 2.1.31). This suggests that VBHC implementation must go beyond technical frameworks and actively promote a collaborative culture. Finally, measuring and evaluating value across the entire care chain was identified as a foundational yet underdeveloped aspect. Stakeholders stressed the need to define what should be measured and ensure consistent interpretation. One provider articulated this clearly, “*VBHC presupposes payment for value generation… alignment can be done by measuring the value input by each actor and ensuring that financing flows follow this allocation*” (Provider 2.4.37). However, others flagged the difficulty in engaging clinicians in this measurement process, calling it the “*crux of the issue*” (Supplier 2.7.1), thereby emphasizing the role of front-line professionals in driving real transformation.

In parallel, the interviews uncovered how stakeholders perceive VBHC as a mechanism to generate competitive advantages across six dimensions. First, differentiation emerged as a key strategy, with organizations viewing VBHC as a way to stand out through superior patient experiences and outcome-driven services. A payer noted, “*I think differentiation is made by the experience you can provide to a patient*” (Payer 3.12.28). Second, evidence of results was widely recognized as a distinguishing feature of VBHC. Stakeholders indicated that demonstrating measurable improvements in patient outcomes could solidify market positioning. As one provider put it, “*The fact that you can have a real vision and real evidence of results…will certainly give a competitive advantage*” (Provider 3.3.26). Third, the theme of pursuing continuous improvement highlighted how VBHC fosters iterative development. Stakeholders described it as a catalyst for operational learning and innovation. “*It’s easier to start changing and leveraging improvements in areas that are not so developed*,” one provider stated (Provider 3.3.37). Fourth, transparency was seen as both a cultural shift and a strategic asset. “*We must learn to show others what we did well…what gain we obtained*” (Consumer 3.1.26), emphasized a patient representative, underlining the role of transparency in building credibility and trust. Fifth, stakeholders connected VBHC to more efficient access to resources and financing, especially in a system where funding is increasingly tied to demonstrable performance. “*Institutions compete for more funding…in specialties that have VBHC projects implemented*,” observed a payer (Payer 3.10.27). Finally, competitiveness and reputation were considered by several participants as natural byproducts of VBHC engagement. As one supplier noted, “*There is the reputational aspect that is relevant*” (Supplier 3.6.7), suggesting that visibility in VBHC initiatives strengthens stakeholder positioning.

These findings must be understood within the broader structure of the Portuguese healthcare system, which is predominantly public, hierarchical, and centrally managed under the Serviço Nacional de Saúde (SNS). While this structure allows for uniform policy and the potential scalability of VBHC initiatives, it also presents rigidities in budgeting, fragmented digital infrastructure, and limited flexibility for local experimentation. The system remains oriented around provider-centric incentives and administrative compliance, rather than outcome-based evaluation or patient-centric care. This misalignment poses challenges for full VBHC adoption and underscores the importance of targeted policy reform and cross-sector engagement.

Ultimately, this study bridges the theoretical promises of VBHC with the lived realities of its implementation in Portugal. While the conceptual appeal of value creation through stakeholder alignment, outcome measurement, and transparency is widely acknowledged, the findings illustrate that these ambitions must contend with deep-rooted systemic and cultural challenges. The lack of interoperable data systems, limited clinician engagement, and fragmented stakeholder coordination all represent structural barriers that require deliberate policy action. At the same time, stakeholders identified clear strategic opportunities—such as differentiation, improved resource access, and reputation building—that can incentivize adoption if properly aligned with reform initiatives. These insights suggest that for VBHC to succeed in Portugal, implementation must be guided by a dual focus: strengthening foundational infrastructure (data, trust, and standardization) while leveraging competitive incentives to drive stakeholder engagement. In doing so, VBHC can move from aspiration to action, contributing meaningfully to the transformation of Portuguese healthcare.

### Strengths and Limitations

This is the first study in Portugal to focus on the perceptions of stakeholders regarding VBHC. The in-depth qualitative analysis provides rich, contextual insights into multi-stakeholder alignment and VBHC as a competitive advantage. Another strength lies in the opportunity to capture diverse perspectives across the healthcare value chain, which strengthens the findings and their relevance to the Portuguese healthcare system.

The sample captures a broad spectrum of institutional perspectives relevant to the research objectives. We acknowledge that the non-random sampling approach may entail selection bias; however, the primary aim was to capture rich, contextualized insights rather than statistical generalization. Thematic saturation was achieved during the analysis, supporting the adequacy of the sample size for this qualitative research.

Regarding limitations, first, the qualitative nature of the study limits the generalizability of the results to a broader context, since a convenience sample of 15 stakeholders may not fully represent all viewpoints within the healthcare sector, and the study’s findings could be subject to interpretation bias, despite efforts to mitigate this through coding validation.

Further research is needed to expand on these findings, including quantitative research that can assess VBHC implementation outcomes on a larger scale. Longitudinal studies could provide insights into the evolving perceptions of stakeholders over time and examine how sustained VBHC efforts impact both patient outcomes and healthcare system efficiency. Additionally, cross-country comparisons could enrich the understanding of VBHC implementation in different healthcare systems.

## 5. Conclusions

This research highlights the critical importance of VBHC as a transformative strategic model for the Portuguese healthcare system. VBHC is positioned as a solution to address the dual challenge of improving patient outcomes while controlling costs. The study reveals that stakeholders in the Portuguese healthcare industry perceive VBHC as a means to create value and gain a sustainable competitive advantage. However, successful implementation hinges on multi-stakeholder alignment, characterized by a common purpose, trust, effective communication, negotiation, and risk sharing. Effective multi-stakeholder alignment in value co-creation requires a shared purpose, trust, open negotiation, and robust communication, along with comprehensive metrics to measure and evaluate value across the entire healthcare value chain. Additionally, adopting VBHC fosters a sustainable competitive advantage through differentiation, evidence-based results, continuous improvement, transparency, and easier access to resources and financing, enhancing both competitiveness and reputation.

## Figures and Tables

**Table 1:** Multi-stakeholder alignment in value co-creation qualitative excerpts, by theme and subtheme.

Theme and Subtheme	Exemplary Quotes
**Establishment of common purpose and trust**	**Provider** 2.3.62 “I consider that a common purpose, an alignment of trust, shared awareness and the capacity for a joint sustainability vision in the balance of the health ecosystem could provide one of the elements of greater involvement of all stakeholders, in addition to a drop in business volume if it focuses on in production for quantity and not value created for the patient.” **Payer** 2.12.1 “I think there is a basic assumption for being able to implement any VBHC strategy, which is to have perfect alignment between stakeholders.” **Consultant** 2.15.32 “The way we define value and the way we measure value, with small nuances, we can achieve very different results. And this creates a huge trust problem between the parties.” **Payer** 2.11.44 “I would say that it will still be a very big challenge to take this step, which is above all also a step of trust among health agents. It is a step that requires a lot of trust between the various agents.” **Consortium** 2.14.17 “The success of this approach will only be achieved if all stakeholders want it, from top decision-makers, i.e., the government, in the case of the public sector, to the most basic element of the teams involved.”
**Availability of negotiation and risk sharing**	**Provider** 2.2.51 “There must be negotiation and compromise.” **Supplier** 2.9.42 “There needs to be a good data platform and good intermediation, we don’t have to agree on everything. As long as we agree on the final end and the moments and forms of data collection that allow me to document that value, I wouldn’t I can question that.” **Provider** 2.3.52 “This strategy of greater shared risk has to be involved in our ecosystem and only then can we move forward together with a better capacity to affect results.” **Payer** 2.10.8 “With contracting and financing, we signal to providers what we want them to do to create value.”
**Effective communication and collaborative momentum**	**Provider** 2.2.52 “Involve all stakeholders from the beginning and ensure clear and effective communication at all phases of the project.” **Supplier** 2.5.19 “Improve communication between different stakeholders that interact in patient management.” **Patients** 2.1.31 “We should be more sensitive to replicating what others do well.” **Payer** 2.11.5 “It’s About having the provider and the payer very well aligned.” **Consultant** 2.15.19 “As long as there is no national alignment strategy here, and once again, this will not happen all at once for all therapeutic areas. What is needed is a kind of consensus, once again, segment by segment.”
**Measuring and evaluating value across the value chain**	**Provider** 2.4.37 “VBHC presupposes payment for value generation, the way to align partners can be done based on measuring the value input by each of them (see what is the most efficient level for producing the same value), and ensuring that the financing model/flow follows this value allocation.” **Supplier** 2.8.8 “I think it is important to understand what you want to measure and whether what you want to measure and the interpretation of those who are going to record it are the same.” **Consortium** 2.14.18 “This issue of measuring and the consequence of measuring, which is being able to compare, this won’t be easy. First of all, we don’t have this endogenized culture”. **Supplier** 2.7.1 “Convincing the Clinician to measure. The Clinic is the big crux of the issue; it is where we have to invest and realize that it is a process that will guarantee the effectiveness of what it uses.”

**Table 2:** Multi-stakeholder alignment in VBHC as a competitive advantage, qualitative excerpts, by theme and subtheme.

Theme and Subtheme	Exemplary Quotes
**Differentiation**	**Payer** 3.12.28 + 3.12.30 “I think that differentiation is made, or competitive advantage is acquired, by differentiating the experience that can be provided to a patient.” **Consortium** 3.14.27 “The pharmaceutical and medical device industry, let’s say, incorporate VBHC into their business model, and in my opinion they will be able to take advantage of this, because this is a way of differentiating.” **Researcher** 3.13.22 “I think it’s a competitive advantage. It may be a differentiating factor, but it is still difficult to find projects in Portugal whose VBHC methodology is implemented from A to Z.” **Consultant** 3.15.23 “We are obligatorily generating information that our competition may not have generated and this will obviously have a positive effect on the stakeholders we are interested in impacting.”
**Evidence of results**	**Provider** 3.3.26 “The fact that you can have a real vision and real evidence of results, appropriate to your costs, will certainly give a competitive advantage to those who only have health insights.” **Supplier** 3.5.28 “Companies that manage to bring to the National Health System this possibility of measuring and managing the quality of their data, of the therapeutic choices they use for their patients, will undoubtedly be companies that will have a competitive advantage in the future.” **Provider** 3.2.37 “It can be seen as a competitive advantage to integrate into a project like this, as it allows the organization to stand out for its offer of services, centered on the patient, with a focus on results, quality, instead of focusing only on processes and in the activities, it is producing.” **Provider** 3.3.39 “Today the only thing we have is a mere perception of our results and not absolute evidence of what our strategic positioning is, so it will necessarily allow a very significant competitive advantage through the evidence of what we are producing.” **Patients** 3.1.27 “I think that when the comparability of success rates between hospitals begins to be revealed in the health area, it will be a differentiating factor for the patient.” **Consortium** 3.14.21 “The use of Value Based Healthcare has not yet reached the level where it allows me to compare units with units, hospitals with hospitals.”
**Pursuing continuous improvement**	**Provider** 3.3.37 “Fundamentally, firstly, because it is easier to start changing and being able to leverage these opportunities towards situations that are not so good, that can undergo these positive progressions. And, secondly, so that this evolution can be done in a timely manner and with progression.” **Supplier** 3.6.8 “We have been able to demonstrate […] that [VBHC] is something we can do, where we can experiment, understand and create new types of collaboration.” **Provider** 3.4.18 “I truly believe in the importance of data and monitoring the results of our interventions to improve processes and understand whether what we are doing is being effective or not.”
**Transparency**	**Patients** 3.1.26 “Through VBHC we have to learn to show others what we did well, what went well, what indicators were achieved, what gain we obtained, what the result was.” **Payer** 3.10.24 “Our health organizations don’t have much incentive for competition. […] Therefore, it is difficult to introduce this issue of competition.” **Provider** 3.3.38 “A different way of looking at the health market and its stakeholder relationships, in a much more transparent way.” **Provider** 3.4.20 [The advantage is] “we have result indicators so we can measure the processes.” **Consultant** 3.15.25 “It creates a reality of understanding that in fact our technology, beyond the claims of effectiveness or what is generated by the data, our technology has value.”
**Easier access to resources and financing**	**Provider** 3.4.25 “The VBHC can be important for the pharmacy sector to be able to attract funding from the National Health Service and from the people themselves and health operators, to prove more efficient than other institutions and health professionals who provide the same service.” **Payer** 3.10.27 “Institutions compete for more funding. In this sense, they are able to have some differentiation, for example, in the specialties that actually have VBHC projects implemented.” **Provider** 3.4.24 “Health institutions and healthcare professionals, we will be competing for the same resources that are finite.”
**Competitiveness and reputation**	**Supplier** 3.7.18 “The medical device industry must compete based on effectiveness.” **Consortium** 3.14.23 “The simple fact that a specific hospital or a specific hospital service is involved in a VBHC project, I see that in itself as being credible.” **Supplier** 3.6.7 “There is the reputational aspect that is relevant.” **Provider** 3.4.23 “If we can do it more efficiently, we will have a competitive advantage.” **Payer** 3.12.37 “If this does indeed bring a specific benefit, I would say that it is a competitive advantage. We believe it is.” **Patients** 3.1.25 “I think so, as long as this is perceived.” **Consultant** 3.15.24 “Beyond the information we generate about certain features of our technology is the perception that we are generating patient-centered value. And that perception is something that very few health technologies can create.”

**Table 3:** Key challenges to implementing VBHC in Portugal.

Challenge	Portuguese Context
Fragmented Digital Infrastructure	Portugal’s healthcare system lacks interoperable IT systems across providers, hindering the collection and sharing of outcomes data critical for VBHC implementation [29,30,31]
Centralized Budget and Limited Flexibility	The National Health Service (SNS—Serviço Nacional de Saúde) operates under a rigid, centralized budgeting system, limiting the autonomy of hospitals and providers to innovate or adapt funding models [29,32,33]
Institutional Silos and Low Trust	Public–private cooperation is often hindered by mistrust and historical silos between healthcare actors, complicating collaboration and data sharing necessary for VBHC [29,32,34]
Limited Outcome Measurement Culture	Portuguese providers often lack incentives and tools to systematically measure outcomes, with current systems focused on activity-based metrics rather than value-based indicators [34,35]
Urban–Rural Inequity	Resource allocation disparities between urban centers and interior regions challenge equitable implementation, risking increased inequality if VBHC is applied uniformly [36,37].

SNS—National Health Service (Serviço Nacional de Saúde); VBHC—Value-Based Healthcare.

## Data Availability

Requests to access the datasets should be directed to the corresponding author and will be granted upon reasonable request.

## Supplementary Materials

The following supporting information can be downloaded at: https://www.mdpi.com/article/10.3390/jmahp13030044/s1, Supplementary Table S1—Research questions analyzed (Interview guide); Supplementary Table S2—Profile and distribution of interviewed stakeholders.
