# Supplementary material for: Value-Based Healthcare as a Competitive Strategy—A Multi-Stakeholder Perception Analysis in Portuguese Healthcare
Source: J Mark Access Health Policy. 2025 Sep 2;13(3):44. doi: 10.3390/jmahp13030044 (PMC12452736; doi:10.3390/jmahp13030044)
Supplement: Supplementary file 1 [file jmahp-13-00044-s001.zip › jmahp-3711249-supplementary.pdf]

Journal of
Market Access
& Health Policy

MDPI

Article – Supplementary Material

Value-Based Healthcare as a Competitive Strategy – A Multi-Stakeholder perception analysis in Portuguese Healthcare

Filipe Santiago [1,*], Filipe Costa [2], Eduardo Redondo [2,3], Cristiano Matos [4]

[1] Medtronic, Lisbon, Portugal; filipesantiago@gmail.com (F.S.)
[2] Nova School of Business and Economics, Lisbon, Portugal; filipe.costa@novasbe.pt (F.C.)
[3] Universidade Europeia, Lisbon, Portugal; eduardoredondo@hotmail.com (E.R.)
[4] Instituto Politécnico De Coimbra, ESTESC-Coimbra Health School, Farmácia, Coimbra, Portugal; cristiano.matos@estesc.ipc.pt (C.M.)
* Correspondence filipesantiago@gmail.com (F.S.)

Supplementary Table S1 - Research questions analyzed (Interview guide)

What do you mean by value? Can you give an example?
Given that the perspectives of the different stakeholders are potentially different from each other, what do you consider necessary for all stakeholders to be aligned for the proper and successful implementation of VBHC projects?
Integration into a VBHC project is a potential competitive advantage by differentiation. As a VBHC stakeholder, what is your perception of this statement? Can you share any examples?
How do you see the future of VBHC as a strategic model in your sector?
How can patient-centered healthcare create value?
In your opinion, how can you balance a high-quality care offer with the need to reduce costs in the health system? What are the current priorities to ensure this balance in the future?
As a VBHC stakeholder, what are your most important contributions in the eventual implementation of VBHC projects?
As a VBHC stakeholder, what are your most important challenges in the eventual implementation of VBHC projects?
In addition to the issues addressed, do you consider any additional issues that could be considered in this research?
Do you recommend any stakeholder/professional who can contribute to this research?

VBHC – Value-Based Healthcare

Supplementary Table S2 - Profile and distribution of interviewed stakeholders

Participant ID	Stakeholder Type	Organization Type	Nationality	Sex	Role
**1**	Consumer (Patient Association)	Patient Association	Portuguese	F	President
**2**	Public Healthcare Provider	Public Hospital	Portuguese	F	Data Analyst
**3**	Private Healthcare Provider	Private Hospital	Portuguese	M	VBHC Director
**4**	Pharmacies	Pharmacy	Portuguese	M	President
**5**	Supplier (Medical Devices)	Medical Devices Company	Multinational	F	Commercial Manager
**6**	Supplier (Medical Devices)	Medical Devices Company	Multinational	M	VBHC Director
**7**	Supplier (Medical Devices)	Medical Devices Company	Multinational	M	General Manager
**8**	Supplier (Pharmaceuticals)	Pharmaceutical Company	Multinational	F	Commercial Director
**9**	Supplier (Pharmaceuticals)	Pharmaceutical Company	Multinational	M	Innovation Director
**10**	Public Payer	Public Payer	Portuguese	F	Innovation Coordinator
**11**	Private Payer	Private Payer	Multinational	M	Strategic Manager
**12**	Private Payer	Private Payer	Multinational	M	Coordinating Director
**13**	Academic Researcher	Academic Institution	Portuguese	M	Economist
**14**	Consortium Representative	Healthcare Consortium	Portuguese	M	President
**15**	Consultant	Multinational Consulting Firm	Multinational	M	Innovation and Value Creation Consultant

VBHC – Value-Based Healthcare
